# Mechanisms of nonalcoholic fatty liver disease and implications for surgery

**DOI:** 10.1007/s00423-020-01965-1

**Published:** 2020-08-24

**Authors:** Benedikt Kaufmann, Agustina Reca, Baocai Wang, Helmut Friess, Ariel E. Feldstein, Daniel Hartmann

**Affiliations:** 1 Department of Pediatric Gastroenterology, University of California San Diego (UCSD), La Jolla, CA USA; 2 Department of Surgery, TUM School of Medicine, Klinikum rechts der Isar, Technical University of Munich, 81675 Munich, Germany

**Keywords:** NAFLD, Liver, Obesity

## Abstract

**Background:**

Nonalcoholic fatty liver disease (NAFLD) has become the most common form of chronic liver disease in both adults and children worldwide. Understanding the pathogenic mechanisms behind NAFLD provides the basis for identifying risk factors, such as metabolic syndrome, pancreatoduodenectomy, and host genetics, that lead to the onset and progression of the disease. The progression from steatosis to more severe forms, such as steatohepatitis, fibrosis, and cirrhosis, leads to an increased number of liver and non-liver complications.

**Purpose:**

NAFLD-associated end-stage liver disease (ESLD) and hepatocellular carcinoma (HCC) often require surgery as the only curative treatment. In particular, the presence of NAFLD together with the coexisting metabolic comorbidities that usually occur in these patients requires careful preoperative diagnosis and peri-/postoperative management. Bariatric surgery, liver resection, and liver transplantation (LT) have shown favorable results for weight loss, HCC, and ESLD in patients with NAFLD. The LT demand and the increasing spread of NAFLD in the donor pool reinforce the already existing lack of donor organs.

**Conclusion:**

In this review, we will discuss the diverse mechanisms underlying NAFLD, its implications for surgery, and the challenges for patient management.

## Introduction

Nonalcoholic fatty liver disease (NAFLD) is the leading primary cause of chronic liver disease in both children and adults worldwide today. Global prevalence is estimated at 25% and accounts for the most common etiology of abnormal liver function tests in western countries [1]. NAFLD has reached epidemic proportions yet important geographic variabilities must be recognized. Its prevalence has been reported to be the highest in South America and the Middle East (> 30%), followed by Asia (27%) and North America and Europe, who have shown similar prevalence rates (24% and 23%, respectively) [2]. Ethnicity also accounts for discrepancies within regions that may be explained by differences in lifestyle and diet, access to health care, and genetic factors. In the USA, Hispanic Americans have the highest prevalence of NAFLD and it has been shown that ethnic backgrounds influence individual susceptibility to the development of this disease [2].

NAFLD encompasses the entire spectrum of disease which are characterized by hepatic steatosis that develops in the absence of competing liver disease etiologies such as alcohol consumption, monogenic hereditary conditions, or iatrogenic causes [3]. Excessive triglyceride accumulation in hepatocytes defines NAFLD but the presence of additional histological abnormalities is a key factor in the stage of disease, progression, and outcomes. Nonalcoholic steatohepatitis (NASH), an inflammatory stage of NAFLD, is characterized by steatosis as well as lobular inflammation and hepatocyte injury [4]. It represents a more severe course of the disease resulting in a higher risk of progressing to severe fibrosis, subsequently cirrhosis and hepatocellular carcinoma (HCC) [2]. Although the degree of fibrosis and scar formation is independent and varies in every stage of NAFLD, it represents the main prognostic factor for long-term outcomes and liver-related mortality in this subpopulation [1].

NAFLD prevalence is rising in parallel with the worldwide increase of obesity and insulin resistance. A constellation of metabolic disorders is usually present in patients with NAFLD and includes obesity, type 2 diabetes mellitus (T2DM), dyslipidemias, and other features of the metabolic syndrome (MS). The prevalence in patients undergoing bariatric surgery exceeds 90%, and it is diagnosed in 55% of patients with T2DM [3, 5]. The same metabolic risk factors that trigger cardiovascular disease (CVD) appear to underly the development of NAFLD. Consistent with this finding, cardiac and liver-related complications are the leading causes of morbidity and mortality in this population [4]. The presence of metabolic comorbidities and cardiovascular disease has significant clinical implications for the management and treatment of the disease. Although patients usually have typical features of MS, it is increasingly recognized that NAFLD can develop in the absence of obesity. Lean NAFLD, diagnosed in patients with BMI < 25, may be present in up to 10–20% of the American and European population and deserves the same clinical attention [2, 6].

Given the rise in metabolic diseases and the consideration of new dietary and lifestyle patterns, a sustained increase in NAFLD prevalence is expected. It is estimated that 20% of patients develop NASH, a stage within the spectrum that is associated with higher mortality rates [4]. The progression of the disease leads to increased liver-specific and life-threatening complications, such as cirrhosis and HCC, which represent an enormous burden for the future. The upcoming challenges and demands on health care are even greater since it has been shown that non-cirrhotic NASH patients can also develop HCC [3]. Accordingly, the burden of NAFLD-related liver transplantation (LT) has increased dramatically and places a growing strain on the health system. While NAFLD-associated HCC is increasing drastically, NASH-cirrhosis is now considered the second most common indication for LT in the USA after chronic hepatitis C [2]. It is important that the complexity of these patients is taken into account when considering and evaluating patient management. While NAFLD prevalence remains undiminished, its clinical, economic, and health implications are undeniable: NAFLD has slowly become the leading cause of chronic liver disease, patients usually coexist with multiple metabolic complications, and it will soon emerge as the primary indication for liver transplantation.

## Current and future implications of pediatric NAFLD

The global obesity epidemic of the twenty-first century has led to a dramatic increase in the rate of obesity among children [2]. The exponential risk of developing obesity-related liver disease in this population poses an enormous burden on social care and health systems in the near future. It is estimated that NAFLD affects 3–10% of the general pediatric population and it has emerged as the most common cause for chronic liver disease in children. The intake of high caloric foods in combination with a sedentary lifestyle foreshadows the onset of fatty liver disease. Central obesity along with insulin resistance represents the strongest risk factors for childhood NAFLD. Despite its strong association with an unhealthy lifestyle and weight gain during school years, the complex interplay of several environmental factors in a genetically susceptible individual must be taken into account. Further elucidation of the pathophysiology behind the development and progression of NAFLD in children will help to identify susceptible individuals, trigger factors, and modifiable lifestyle changes. In combination, this will hopefully contribute to early diagnosis, prevention, and patient-tailored therapy [7].

Children with NAFLD have multiple obesity-related comorbidities, such as insulin resistance, dyslipidemia, MS, and obstructive sleep apnea. Yet, few or no clinical signs and symptoms of NAFLD hinder an early diagnosis. Patients are usually asymptomatic, and diagnosis is often incidental at a mean age of 11–13 years. In addition, current diagnostic techniques lack sensitivity and specificity to determine the stage of disease and the extent of liver fibrosis. The progression from reversible conditions such as steatosis to irreversible cirrhosis makes early diagnosis a key element for proper and timely treatment [7].

The long life expectancy of children corresponds to the increased risk of developing long-term complications related to NAFLD. However, further studies are needed to examine the exact outcomes that result from childhood NAFLD. Weight gain in childhood and adolescence not only is associated with NAFLD development but also makes individuals susceptible to end-stage liver disease and HCC later in life [7]. With the earlier onset of the disease, associated complications will appear earlier, and clinicians and surgeons are faced with younger patients who need treatment. Poor quality of life, shorter life expectancy, and a burden on the health system will reflect the expectation of end-stage liver disease and HCC [2].

## Mechanisms of NAFLD/NASH

**Fig. 1:**
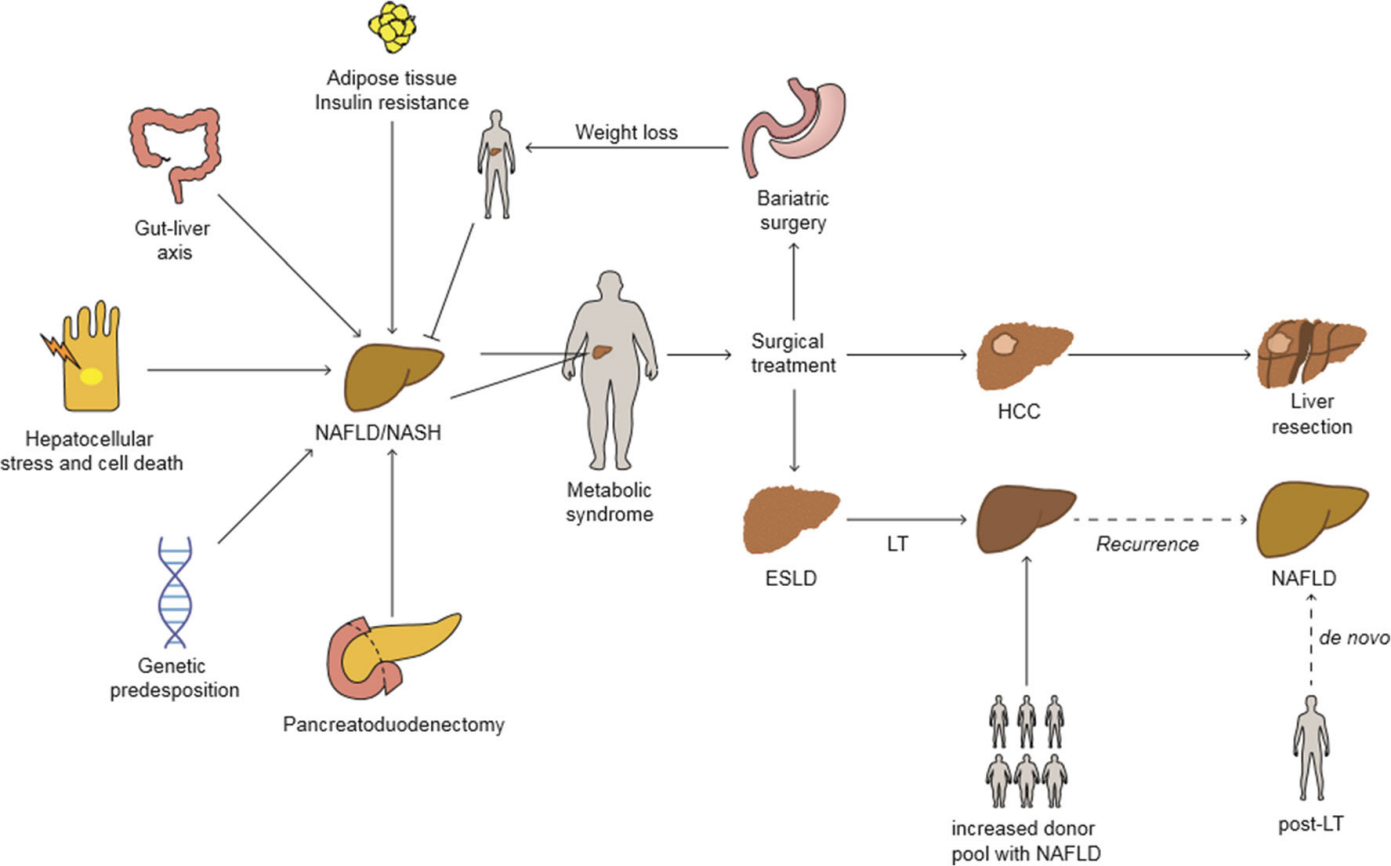
Mechanisms of NAFLD/NASH and surgical treatment strategies. The pathogenesis of NAFLD is complex and multifaceted. NAFLD development is closely related to the presence of the metabolic syndrome (MS). In this scenario, obesity-associated inflammation and insulin resistance were identified as key contributors to the disease. Lipotoxic-induced cell stress and hepatocyte cell death fuel NAFLD and promote the transition to more severe stages, such as NASH. Changes in the gut microbiota as well as in the host’s genetics modify an individual’s susceptibility to disease development and progression. In particular, the setting of exocrine pancreatic insufficiency and malnutrition caused after pancreatoduodenectomy has also been proven to be an important factor favoring the development of NAFLD. The intrinsic relationships between MS, NAFLD, and obesity is well known. While weight loss from lifestyle changes remains the primary recommendation, barriers to such changes along with disease severity make management of NAFLD/NASH by surgery an optimal treatment. However, the coexistence of multiple metabolic comorbidities in patients with NAFLD carries an increased risk when considering surgery. Bariatric surgery has become an alternative to achieving significant weight loss and has been shown to stop NAFLD progression. Liver surgery is the only curative treatment for HCC and ESLD, two common liver-specific consequences of NAFLD. While liver resection can cure HCC, severe cirrhosis with liver failure requires LT. NAFLD is emerging as the leading indication for LT, and the growing demand poses numerous challenges for patient access to LT and the pre- and post-operative management of patients. The increasing prevalence of NAFLD in the population and the liver donor pool represents a burden for the future. The increasing demands on LT in combination with an excess of NAFLD in the liver donor pools exacerbate the already existing shortness of graft organs. LT liver transplantation, HCC hepatocellular carcinoma, ESLD end stage liver disease, NAFLD nonalcoholic fatty liver disease, NASH nonalcoholic steatohepatitis

The development of steatosis as well as the progression to NASH and fibrosis represents a complex and dynamic process (Fig. 1). A combination of environmental factors, host genetics, and gut microbiota can lead to an excessive influx of free fatty acid (FFA) influx and accumulation of triglyceride (TAG) in the hepatocytes, creating a lipotoxic environment that leads to hepatocyte cell death, liver inflammation, fibrosis, and pathological angiogenesis. The subsequent hepatic inflammatory response promotes fibrogenesis in the liver and is an important driving force for disease progression. Several factors work in parallel or sequentially to promote sustained inflammation, hepatocellular injury, and an abnormal wound healing response shaping the clinical and histological features of the disease [8, 9].

## Insulin resistance and adipose tissue inflammation

The coexistence of multiple metabolic comorbidities in patients with NAFLD is widely recognized and reflects systemic dysfunctional metabolic pathways. Obesity and insulin resistance form the basis for the development of hepatic steatosis. Obesity is known to be associated with a low-grade inflammatory state in adipose tissue (AT) that alters cytokine secretion and impairs insulin signaling [1, 10]. Obesity-related insulin resistance (IR) hinders TAG storage, leads to uncontrolled AT lipolysis, and consequently results in high levels of circulating FFA. An increased hepatic influx of FFA fuels TAG synthesis, and at the same time, insulin resistance paradoxically upregulates de novo lipogenesis (DNL) in hepatocytes, which further promotes hepatic fat accumulation [8]. Dietary sugars and fats are also important sources of TAG synthesis that contribute to hepatic steatosis [1]. In turn, the buildup of lipids in the liver alters its endocrine function and contributes to the maintenance and deterioration of IR. Disrupted protein signaling from the liver in the setting of NAFLD affects inflammation, lipogenesis, and fatty oxidation pathways in the liver, AT, skeletal muscle, and in the pancreas, all of which regulate insulin action [11]. IR and NAFLD consequently enter a vicious progression cycle and regulate each other positively. Understanding the relationship between these metabolic comorbidities is therefore essential, especially when considering patient management.

## Hepatocellular stress

Oxidative stress is involved in the pathophysiology of chronic liver disease. Liver damage is mediated by direct cellular injury, cell degeneration and demise via apoptosis, and other forms of cell death, proinflammatory cytokine expression, hepatic stellate cell activation (HSCs), and abnormal wound healing responses leading to fibrosis [12]. Oxidative stress results from an imbalance between pro-oxidant species and anti-oxidant defense capacities. The oxidative injury to liver cells is enhanced by an overproduction of toxic lipid metabolites in the setting of hepatic steatosis. Aberrations in the lipid metabolism together with dietary intake of FFA establish a lipotoxic hepatic environment that triggers hepatocellular injury and an inflammatory response which supports the transition from simple steatosis to NASH. The presence of lipid peroxidation markers has been shown to correlate with myeloperoxidase-positive neutrophils, an important source of reactive oxygen species (ROS), and disease severity [13]. Activation of NADPH oxidase, the main enzyme in ROS production, in Kupffer cells (KCs) and HSCs also contributes to inflammatory and fibrogenic signaling in response to noxious stimuli such as danger-associated molecular patterns (DAMPs) and products of lipid peroxidation [9].

Mitochondria are the primary cellular source of ROS and therefore a potential determinant for hepatocellular injury. Patients with NASH have demonstrated to have defective hepatic ATP synthesis due to a reduced mitochondrial respiratory chain activity [14]. ROS-mediated mitochondrial DNA damage and mitochondrial membrane lipid peroxidation accumulate over time and lead to cellular dysfunction. Consistent functional and structural mitochondrial aberrations can in turn potentiate mitochondrial oxidative damage, lead to loss of mitochondrial integrity, and trigger proapoptotic signaling pathways, all of which contribute to disease progression [12].

## Hepatocellular death

Cell death is the ultimate driver of liver disease development and fibrosis progression. Despite its well-known function in maintaining homeostasis in the healthy liver, chronic liver diseases that promote tissue fibrosis, cirrhosis and HCC, trigger a maladaptive response to cell death. Hepatocyte ballooning, apoptosis, necro-inflammation, and different degrees of fibrosis in the setting of hepatic lipid accumulation histologically characterize NASH, indicating the occurrence of multiple types of cell death [15]. Different modes of cell death such as apoptosis, necrosis, necroptosis, and pyroptosis have been shown to contribute to liver disease in a cell-, stage-, and context-specific manner [16].

### Apoptosis

Apoptosis is a well-known and highly regulated form of cell death. Although usually considered a silent and noninflammatory process, apoptosis has been identified as an important mechanism for liver damage and is strongly associated with the pathogenesis of NAFLD [16]. The extent of hepatocyte apoptosis correlates with disease severity, and patients with NASH have demonstrated to have a stronger expression of several apoptotic pathways compared with those with only hepatic steatosis. Feldstein et al. have demonstrated active caspases 3 and 7 as well as a strong expression of Fas receptors in NASH specimens, all which confirm the occurrence of apoptosis in this disease [17]. A causal relationship between apoptosis and liver fibrosis has also been elucidated. Phagocytosis of apoptotic hepatocytes by quiescent hepatic stellate cells stimulates their fibrogenic activity and leads to collagen accumulation, which translates into fibrosis and posterior cirrhosis [18]. Hepatocyte apoptosis has also been linked to the release of mediators that promote a cellular immune response and create an inflammatory milieu.

A wide range of intrinsic and extrinsic cellular events functions as triggers of apoptosis, and mechanistically, the activation of caspases, Bcl-2 family proteins, and c-Jun N-terminal kinase-induced hepatocyte apoptosis play a role in the progression of NASH [18]. The increased influx of fatty acids has a lipotoxic effect on hepatocytes, and lipid-induced apoptosis, a process termed lipoapoptosis, promotes fibrosis in the setting of liver steatosis. Caspase-2 is an initiator-caspase in lipoapoptosis, and its activity enhances pro-fibrogenic events [18]. Death receptor-mediated apoptosis is also a key feature of liver disease and has an inflammatory effect through the secretion of extracellular vesicles and chemokines that recruit and activate immune cells [16]. The Fas receptor is upregulated in steatotic livers, and TRAIL-receptor signaling correlates with liver injury, macrophage-associated inflammation, and fibrosis [17, 19]. Hepatocyte-apoptosis plays a fundamental role in the progression from simple, “benign” steatosis to NASH. However, a recent study using the pan-caspase inhibitor emricasan in NASH patients failed to improve disease activity and fibrosis and suggested that inhibition of the main apoptotic caspases 3 and 7 resulted in a shift to other types of cell death [20].

### Necroptosis

Necroptosis is a regulated type of cell death that includes features of apoptosis and necrosis. It is believed that it acts as a backup mechanism of programmed cell death when apoptosis is inhibited. Necroptosis shares upstream activating mediators with apoptosis but morphologically resembles necrosis as it causes cell swelling and rupture [16]. Receptor interacting proteins (RIPs) are important cell stress sensors that activate the necroptotic pathway, and the formation of the “necroptosome,” a complex containing RIPK1, RIPK3, and mixed lineage kinase domain-like protein (MLKL), leads to membrane lysis and pore formation. The cellular release of DAMPs due to cell membrane disruption is associated with the possible activation of the innate immune system and neighboring cells [21].

Clear understanding of the role of necroptosis in liver pathology remains limited. The lack of expression of RIPK3 in hepatocytes under basal conditions is comparable with the increased expression that was observed after chronic ethanol feeding and dietary mouse models of steatohepatitis. Upregulation of RIPK3 has also been demonstrated in human NASH and in patients with alcohol liver disease [21]. Although HFD mouse models show an upregulation of RIP3, the genetic deletion of RIP3 increased inflammation and hepatocyte apoptosis, suggesting a protective role of necroptosis in the pathogenesis of NAFLD [22]. This markedly contrasts previous findings that showed the harmful effects of RIP3 on liver injury in response to methionine- and choline-deficient (MCD) feeding. RIP3K-dependent necroptosis in MCD-fed mice led to liver injury, inflammation, induction of hepatic progenitor cells/activated cholangiocytes, and liver fibrosis [15]. The important differences in the pathophysiology between HFD and MCD diet-induced liver injury suggest that the metabolic state of the liver orchestrates the balance between the different types of programmed cell deaths [22]. The controversial findings in the two dietary models support that the different activation of specific types of cell death influences the outcomes in liver disease [23].

### Pyroptosis

Pyroptosis is the most recently described type of programmed inflammatory cell death. Caspase-1, a pro-inflammatory caspase, activation is the primary upstream mechanism of this pathway and involves the assemble of inflammasome multi-protein complexes and the formation of pores in the cell membrane [9]. The resulting maturation and release of interleukin (IL)-1β and IL-18 into the extracellular space and the circulation make this mechanism intrinsically proinflammatory. Studies have shown that active inflammasome particles are also released during this process and suggest pyroptosis as a novel mechanism that transmits inflammatory signal to neighboring cells [21].

Different mechanisms drive pyroptosis and include canonical and noncanonical inflammasome activation [21]. Global NLRP3-overactivated mutant mice have pronounced pyroptotic cell death and severe inflammatory changes and liver fibrosis. This reveals NLRP3-mediated pyroptosis as a harmful hepatic stimulus [24]. Gasdermin D (GSDMD) has recently been recognized as a key effector molecule in pyroptosis-induced cell membrane pore formation but a clear understanding of its contribution according to each type of liver injury is still limited. GSDMD levels are elevated in livers of NAFLD, and expression correlates with disease severity. GSDMD global knockouts had a protective effect when fed a MCD diet and showed less steatosis, inflammation, and fibrosis [25].

## Immune system

The role of immune cells is necessary to maintain organ homeostasis as they act as stress sensors and mediate a balanced and well-coordinated response under physiological conditions. An overactive immune response becomes pathological and is seen in the context of chronic-inflammatory-driven liver disease such as NASH. Pattern recognition receptors in immune cells play a pivotal role in NASH, and downstream signals trigger a cascade of inflammatory cytokine secretion [9]. In addition to other endogenous and exogenous factors, dying hepatocytes release danger signals that induce the activation of a sterile inflammatory response in cells of the innate immune system [26]. Intercellular crosstalk increases pro-inflammatory signaling to neighboring cells, and novel drivers of inflammation are constantly being discovered. Importantly, HSC activation is sensitive to most of these factors. Although activated HSCs intend to mitigate liver damage, persistent activation leads to pathological scar formation and destruction of liver architecture.

In early stages of the disease, KCs coordinate the recruitment of other immune cells and promote hepatocyte apoptosis by releasing IL-1β, TNF, CCL2, and CCL5 [27]. KCs and infiltrating monocyte-derived macrophages represent different subsets of liver macrophage populations that contribute to liver inflammation and damage in different but significant ways. KCs have been shown to promote lipid accumulation, a predominant characteristic of NAFLD [28]. Neutrophil migration to the injured liver is a major source of ROS. Though necessary to alleviate the noxious stimuli, activation of neutrophils must be tightly regulated due to adverse cytotoxic effects. A higher neutrophil-to-lymphocyte ratio was found in patients with NASH and reflects enhanced physiological stress leading to more severe forms of the disease [29]. Innate immune cells represent a dynamic population in the setting of liver injury, and a dysregulated or hyper-stimulated communication between these cells creates an ideal environment for the transition of hepatic steatosis to steatohepatitis and fibrosis.

## Inflammasome

Inflammasomes are intracellular multiprotein complexes present in liver parenchymal and nonparenchymal cells that sense danger signals and control the activation of caspase-1. The downstream cleavage and activation of pro-caspase-1 and the secretion of pro-inflammatory cytokines IL-1β and IL-18 upon stimulation make inflammasomes key regulators of inflammation and cell fate [26, 30]. The IL-1 pathway has been described as an important mediator for the transition from liver steatosis to steatohepatitis and liver fibrosis in murine models of NASH [31]. Inflammasome-induced pyroptosis contributes to an inflammatory feed-forward loop since, as previously described, this cell death mechanism is intrinsically pro-inflammatory. In addition to intracellular activity, the release of inflammasome particles following pyroptosis has been discovered as a novel mechanism that transmits inflammasome signaling to neighboring cells [26].

NLRP3 is the best characterized inflammasome involved in chronic liver injury and contributes significantly to NAFLD development. Constitutive activation of NLRP3 in mouse models exacerbated liver inflammation and highlighted its role in disease progression to steatohepatitis and early fibrosis [32]. Several pathways, including pathogens and sterile insults, have been described to activate NLRP3 and the molecular mechanisms behind them include a decrease of intracellular K+ concentration, the generation of mitochondrial-derived reactive oxygen species (ROS), the rise of cytosolic Ca2+, and the activity of lysosomal proteases [26]. The presence of NLRP3 inflammasome in hepatocytes, HSCs, and liver macrophages has revealed an important crosstalk between all liver cellular types that orchestrate inflammatory signaling. Cell specificity of inflammasome activation has elucidated a particularly important role of NLRP3 activation in HSCs, the main fibrogenic effector cell in liver disease. Aberrant activation of HSCs to myofibroblasts leads to increased production and deposition of extracellular matrix proteins and the resulting scar formation seen in liver fibrosis. NLRP3 inflammasome induction in HSCs favors the switch to myofibroblasts and is sufficient to trigger fibrogenesis regardless of inflammation [33]. These findings support that inflammasomes are key modulators of the main processes that mediate chronic, persistent liver injury.

## Gut-liver axis

The intestinal microbiota (IM) represents a complex ecosystem that is actively involved in protective, immune, metabolic, and trophic functions. The multidirectional interplay between an imbalanced IM, the immune system, the liver, and metabolic pathways is increasingly recognized in the pathogenesis of NAFLD [34].

Obesity is an important determinant of NAFLD, and alterations in the composition of the gut microbiota have been reported in overweight people. The ability of the microbiota to gain energy from food is greater in obese individuals and demonstrates that microbial genome plays a role in determining the obesity prone state [34].

The gut-liver axis includes a close bidirectional communication: (1) the secretion of bile acids and bioactive mediators by the liver into the biliary tract and, (2) the metabolism of endogenous and exogenous substrates by gut microorganisms which translocate into the portal vein in order to reach the liver [35]. Intestinal dysbiosis (imbalanced gut microbial composition) disrupts the crosstalk between the gut and liver by increasing gut barrier permeability, modifying bile acid (BA) composition, and altering enzymatic and metabolic pathways. The loss of gut barrier integrity leads to the translocation of bacteria and bacteria-derived products that mediate liver cell activation through innate immune cell receptors [36]. The subsequent immune response, mainly mediated by toll-like receptors, creates a chronic inflammatory state that contributes to the development of NAFLD.

BA and the gut microbiota have a well-established mutual relationship. On the one hand, the intestinal microbiota controls and modifies BA pool composition through enzymatic activities. On the other hand, BA induce the production of antimicrobial peptides that inhibit gut microbial overgrowth and prevent gut barrier dysfunction. While dysbiosis alters BA composition, BA imbalances also shape IM. BA regulate carbohydrate and lipid metabolism via farnesoid X-activated receptors (FXR), and a shift in BA composition interferes with FXR’s signaling properties. Gut microbiota hence influence NAFLD pathogenesis by regulating the BA/intestinal FXR axis which regulates hepatic lipogenesis [37].

The relationship between choline deficiency and accumulation of hepatic lipids is well known, and dysbiosis-related alterations in choline metabolism and bioavailability contribute to NAFLD development. Host genetics can predispose to choline-utilizing microbiota and together with an imbalance between choline’s metabolites (phosphatidylcholine (lecithin) and methylamines), hepatic triglyceride accumulation, and liver damage [38]. Increased alcohol-producing bacteria in NASH microbiomes has revealed a role of alcohol-producing IM in liver inflammation. The proposed mechanisms include metabolite-induced oxidative stress, weakened intestinal tight junctions, and a resulting inflammatory immune response [35, 39]. A higher prevalence of intestinal dysbiosis in patients with NAFLD and the metabolic effects created by shifts in host’s microbiome profile underlines the IM as an important determinant of susceptibility to disease development progression [40].

## Genetics

The reasons that explain why some subpopulations or individuals are at increased risk of developing a more advanced form of the disease, such as cirrhosis or HCC, remain unclear. There is increasing amount of data showing that genetic interindividual variations influence the development of NAFLD. Single nucleotide polymorphisms (SNP) in genes involved in inflammation, oxidative stress, fibrogenesis, and metabolic pathways have been associated with disease severity phenotypes. Importantly, SNP in two genes, PNPLA3 (encoding patatin-like phospholipase domain-containing protein 3) and TM6SF2 (encoding transmembrane 6 superfamily member 2), are consistently associated with NAFLD [2, 41]. The PNPLA3 rs738409 gene polymorphism is more common in non-obese/lean NAFLD than in non-obese controls [6]. The presence of this allele also correlates with ethnic differences seen in NAFLD, and the findings suggest that it may be a critical element for NAFLD pathogenesis in Hispanics [1, 42]. There is a positive relationship between the PNPLA3 variant (rs738409 c.444C > G,p.Il148M) and hepatic lipid accumulation, steatohepatitis, fibrosis, and HCC. The severity of histological features was linked to the PNPLA3 rs738409 genotype, independent of other risk factors (43). The pathophysiology by which PNPLA3 contributes to NASH progression, and HCC development is not fully understood, but changes in lipid mobilization and aberrant HSCs activation have been identified using various experimental models [8].

## NAFLD following pancreatoduodenectomy

Pancreatoduodenectomy (PD) is the standard surgical treatment for periampullary and pancreatic head tumor lesions, including pancreatic and biliary malignant, premalignant, and benign neoplasms. Advances in surgical techniques, perioperative management, and adjuvant chemotherapy have reduced surgery-related morbidity and mortality [43, 44]. With improved long-term survival rates, there has also been an increase in rates of long-term metabolic complications, including NAFLD. The incidence of NAFLD/NASH following PD is estimated to range from 7 to 40% and it has been shown to develop even in the absence of preoperative MS [45–47]. Multivariable analysis of risk factors showed that female sex, younger age, pancreatic head cancer, and small remnant pancreatic volume were directly related to the occurrence of NAFLD after PD [45, 48, 49]. The literature points to exocrine pancreatic insufficiency (EPI) as the main contributor to post-PD NAFLD. The underlying pancreatic disease, the surgical procedure performed, and the extent of tissue loss are prognostic factors for the development of EPI. EPI is reported in 50–100% of oncological patients’ post-PD as opposed to 0–42% in distal pancreatectomy [50]. Pancreatic head cancer is a constant risk factor for de novo post-PD NAFLD, and it is conceivable that it is due to a small and atrophic remnant pancreas following surgery, which increases the risk of EPI exponentially [45]. Luu et al. showed that patients who underwent pancreatectomy for benign noninvasive intraductal papillary mucinous neoplasms had a lower risk of developing NAFLD compared with patients with malignant disease. PD and the development of pancreatic atrophy were identified as significant risk factors in this study [51]. A small retrospective cohort study found that newly emerging NAFLD only developed occasionally after pancreatic neuroendocrine tumor resection and was mainly observed in patients with recurrent diseases. In contrast to current data, no connection with EPI was found [46]. NAFLD after PD was more common in women with a decrease in serum copper and was also affected by EPI-related malnutrition [43, 44]. Early diagnosis and treatment of NAFLD is important because patients are at increased risk of developing hepatic and cardiovascular life-threatening comorbidities. Beyond the negative effects on the quality of life, functional status, and survival rates of patients, it also leads to additional medical problems and affects possible postoperative treatments including adjuvant chemotherapy [47].

Patient follow-up after PD should be carefully performed as NAFLD can develop in early and late postoperative periods [49]. The etiology, the course of disease, and the clinical manifestations of NAFLD are different in a post-PD setting compared with conventional NAFLD. Obesity and insulin resistance are not predominant in this scenario. Instead, inadequate digestion, absorption, and pancreatic exocrine function dominate the pathophysiology of NAFLD after pancreatic surgery [43, 44]. Theories include malabsorption of amino acids or fat-soluble nutrients, which promote triglyceride accumulation in the liver and an increased conversion of carbohydrates into fat [50]. Steatogenic mechanisms in the post-PD liver include increased FFA uptake and lipogenesis, upregulation of PPARγ, and impairment of VLDL export from the liver [52]. Pancreatic enzyme supplementation has been shown to have positive effects on hepatic steatosis, supporting the strong association between de novo NAFLD and malnutrition due to EPI [53]. The clinical diagnosis of EPI is not sensitive, and clinicians should not simply rule it out for lack of symptoms. Similarly, patients taking enzyme supplementation should be educated about nutrition-related complications to avoid medication non-adherence and its related metabolic complications. Kishi et al. showed that using pancrelipase, an agent that contains several digestive enzymes including lipase, protease, and amylase, was more effective compared with using other digestive enzymes [53]. Nagai et al. demonstrated that pancrelipase improved clinical symptoms, liver function tests, and the nutritional state in patients with previously diagnosed NAFLD [47]. Yamazaki et al. investigated the effectiveness of early administration of pancrelipase via a feeding tube or by mouth of pancreatic enzymes in combination with an elemental diet of branched chain amino acids (BCAA) after PD. A reduced number of patients develop NAFLD in the treatment group vs historical controls [54]. Still, studies have not shown whether stopping enzyme supplementation leads to a refractory appearance of fatty liver [50]. It should be noted that not all patients receiving pancreatic enzyme supplementation were protected from NAFLD, and spontaneous NAFLD regression was observed in patients without treatment. Sato et al. identified a thinner preoperative main pancreatic duct, a lower amylase level, and a higher minimum hepatic CT value, which reflects liver attenuation and depicts the presence of steatosis, to predict of recovery from NAFLD. The presence of these factors should guide the administration of any pancreatic enzyme drug [49]. Adjusting optimal treatment for patients following PD requires uncovering other underlying mechanisms that promote NAFLD occurrence and predisposes paradoxical hepatic fat accumulation in a malnourished state [43, 44].

The presence of NAFLD/NASH is an important risk factor for cardiovascular and liver-specific complications. Patient’s susceptibility to developing NAFLD after PD is exponential and underlines the importance of considering the possibility of EPI and periodically monitoring liver function after surgery. The presence of fatty liver disease and a state of malnutrition following PD contribute to the patient’s morbidity and mortality and are important factors to be considered in patient follow-up.

## Implications for surgery

The increasing number of patients with NAFLD affects both liver and non-liver-specific surgery. Knowledge of NAFLD/NASH is critical for assessing peri-/postoperative morbidity and mortality during major surgery. For liver surgery, in particular, NAFLD/NASH is a serious precondition that must be taken into account when planning treatment strategies. In certain cases, surgery may need to be adjusted based on the condition of NAFLD and may differ from guidelines for patients without NAFLD. Because NAFLD is primarily associated with MS, patients with NAFLD typically have the general, mainly cardiovascular, risk factors for surgery associated with MS. However, even without MS, there is a higher perioperative risk in patients with NAFLD. NAFLD per se is an independent risk factor for cardiovascular disease, which is one of the main comorbidities and the main cause of death in patients with NAFLD. Since cardiovascular events are a common complication after major surgery, the diagnosis of NAFLD must be carefully considered, especially with regard to cardiovascular preoperative evaluation [55–59].

When performing a liver resection, NAFLD is a burden for serious postoperative complications and liver failure [60–62]. After liver resection, the remaining liver volume must be sufficient to ensure adequate liver function. Hereby, the liver’s unique ability to regenerate plays an important role in this [63]. In case of various surgical procedures, in particular in case of oncological tumor resection, the remaining liver volume defines whether a patient can undergo a liver resection or not. A pre-diseased liver affects the ability to regenerate. Dysfunctional lipid metabolism, a characteristic in NAFLD, hinders liver regeneration. Various in vivo studies of partial hepatectomy and liver resection have shown that NAFLD reduces liver regeneration capacity and proliferation [61, 62, 64–67]. It is, therefore, important to carefully assess NAFLD in the preoperative setting. In the context of a pre-diseased liver, this allows an accurate assessment of a suitable remaining liver volume which ensures adequate hepatic function after liver resection.

## Liver resection

Malignant tumors, mainly hepatocellular carcinoma (HCC), are the main indication for liver resection. While alcoholic liver disease and chronic hepatitis infection (hepatitis B-/C-Virus (HBV/HCV)) have been the main risk factors for HCC in recent decades, NAFLD/NASH is a major cause for HCC worldwide today. Since the pathogenesis of HCC due to NAFLD/NASH differs from hepatitis virus and alcohol-associated HCC, a higher prevalence is observed in women and patients that are older when diagnosed with NASH-associated HCC compared with non-NAFLD related HCC [68, 69]. Nevertheless, the risk of developing HCC in patients with NAFLD is significantly higher in men compared with women [70]. Interestingly, ethnicity is also associated with the risk of developing HCC from NAFLD. In a retrospective study in the USA among NAFLD patients, Hispanics showed the highest incidence of HCC. Notably, 20% of HCC cases arising from NAFLD showed no signs of cirrhosis [71]. The increasing numbers of NAFLD-associated HCC and the complexity of these patients raise doubts as to whether perioperative morbidity, mortality, and overall survival vary depending on the etiology of HCC. Studies have shown no difference in reported perioperative morbidity and mortality between HCC caused by either NAFLD, HBV, HCV, or MS [69, 72]. Other studies have shown a higher peri-/postoperative complication rate, including post-hepatectomy liver failure and 30-day mortality, in NAFLD-associated HCC when compared with non-NAFLD-related HCC [61, 73]. In contrast, resection of NAFLD/MS-associated HCC showed favorable long-term survival compared with non-NAFLD-associated HCC [61, 68, 73]. However, a study comparing NAFLD-associated HCC and HBV-associated HCC showed no difference in survival [69]. Interestingly, cirrhotic NAFLD-associated HCC has a similar mortality and survival rate as the non-cirrhotic NAFLD-associated HCC [74]. The data underlines the importance of determining the etiology of HCC for each patient and show a potentially favorable overall survival of NAFLD-associated HCC compared with non-NAFLD-associated HCC after liver resection.

## Liver transplantation

Liver transplantation (LT) is the ultimate treatment for severe liver disease, mainly non-resectable HCC, cirrhosis, and liver failure. In the past two decades, NAFLD has been the fastest growing indication and a major cause of end stage liver disease (ESLD) requiring LT. In the USA, NAFLD is the second leading cause of LT and is expected to be the most common indication for LT in the next two decades. In women, NASH already represents the main cause of LT. While the success of antiviral therapy for HCV has reduced the need for HCV-related LT, the rapidly growing prevalence of NAFLD worldwide, along with the lack of effective therapies, has increased the need for LT in patients with NAFLD [71, 75–78]. Due to the etiology of NAFLD mentioned previously in this review, the characteristics of patients on the waiting list for LT differ from other chronic liver disease. Patients with ESLD related to NAFLD are older, more often female, more obese, and have significantly more metabolic comorbidities (hypertension, hyperlipidemia, type 2 diabetes mellitus) and chronic kidney diseases [71, 75, 78]. The commonly used model for end-stage liver disease (MELD) score indicating the severity of liver disease and the urgency of LT differs between patients with ESLD-related to NAFLD and non-NAFLD. In general, the MELD score of patients on the waiting list due to NAFLD/NASH-related ESLD is lower compared with non-NAFLD ESLD. As a result, patients on the waiting list with NAFLD/NASH-related ESLD have less chance of receiving LT. In addition, while on the waiting list, patients with NASH/NAFLD have a less dramatic deterioration of the MELD score each year which reduces their likelihood of receiving a LT when compared with HCV-related ESLD. In addition, due to their comorbidities, these patients have a higher dropout rate from the waiting list over time. In contrast, similar LT rates were found in patients with a MELD score > 15 regardless of the underlying cause of ESLD (NAFLD or non-NAFLD) [79, 80].

## Impact of NAFLD/NASH on donor livers

The presence of NAFLD not only affects LT recipients but also affects the availability of donor organs. The increasing prevalence of NAFLD worldwide will lead to an increased proportion of both deceased and living liver donors with NAFLD. NAFLD in donors is a burden on the results after LT and may affect donor eligibility. The increasing numbers of potential donors with NAFLD are burdening the already existing lack of liver donors. To date, there are no detailed guidelines that determine whether and to what stage donor livers with steatosis can be used for transplantation. The presence of steatosis is associated with primary non-function, postoperative complications, and a reduction in 1-year graft survival. The current consensus recommends the safe use of liver grafts with mild macrosteatosis (< 30%). The use of livers with moderate graft steatosis (30–60%) is controversial. Traditionally, moderate graft steatosis has been identified as an independent risk factor for graft failure and perioperative morbidity. However, new studies show a reasonable outcome for moderate graft steatosis if the recipients are carefully selected and have no other risk factors. The poor post-transplant outcome associated with severe macrosteatosis (> 60%) in graft livers classified the presence of severe macrosteatosis as a contraindication to LT. However, data suggest that some patients without risk factors and with strict selection criteria may still benefit from a transplant liver with severe macrosteatosis. In general, microsteatosis of any severity is not considered a contraindication to LT [59, 71, 78, 81, 82].

Identifying the grade of steatosis in donors prior to transplantation is an important and challenging aspect. Commonly used imaging modalities such as computed tomography, magnetic resonance imaging, and ultrasonography are not accurate enough in and not sensitive enough to differentiate between micro- and macrosteatosis when predicting steatosis. The gold standard for the diagnosis of steatosis is liver biopsy. However, in the setting of LT, the delay in receiving the result of a liver biopsy extends the cold ischemia time and often makes a liver biopsy an unsuitable tool for NAFLD diagnosis. Newly developed techniques, including magnetic resonance imaging proton density fat fraction (MRI-PDFF) and transient elastography techniques, may overcome the disadvantage of imaging techniques. Recent studies demonstrate that the use of MRI-PDFF to quantify steatosis in living donors and ex vivo on deceased donor livers is a potential alternative and may replace liver biopsy as the golden standard in the future [59, 71, 78, 81–84].

Given the vulnerability of donor organs with NAFLD, there are several methods to preserve the liver during explantation/reimplantation. These methods include hypothermic oxygenated and normothermic machine perfusion as well as venous systemic oxygenated persufflation [71, 82].

Interestingly, steatosis can disappear at a high rate in LT recipients. It has been shown that even recipients of donor livers with a high degree of steatosis show complete resolution within a few months after LT [85]. This supports the hypothesis that graft livers with moderate and severe steatosis could be considered as potential graft organs.

## Outcome of liver transplantation in NAFLD/NASH patients

LT is often the ultimate treatment for patients with cirrhosis, HCC, and ESLD due to various primary liver disease. The growing prevalence of NAFLD and the associated increasing number of NAFLD-related LT raise the question of the outcome of LT in these patients. As discussed in this review, NAFLD patients on the waiting list for LT are more likely to present with obesity, older age, higher cardiovascular risk, and metabolic comorbidities. While the presence of higher peri-/postoperative complication rates in NAFLD patients, mainly cardiovascular events and mortality, remain controversial, long-term postoperative mortality showed no difference between NAFLD/NASH and non-NAFLD/NASH-related LT [57, 59, 78, 86–88]. The short- and long-term results examined in various studies was similar between NAFLD/NASH and non-NAFLD/NASH, including alcoholic liver disease, HBV, cryptogenic cirrhosis, and primary biliary cholangitis/primary sclerosing cholangitis (PBC/PSC). Remarkably, overall survival showed no significant difference [77, 78, 86–90]. Interestingly, post-transplant survival was shown to be higher compared with HCV-associated LT whereas no difference was seen between NASH-associated and alcoholic liver disease-related LT [90, 91]. When comparing the results of HCC related LT, no difference was found between NASH- and non-NASH-associated HCC-related LT. Rather, there was a trend towards a favorable outcome for NASH-associated HCC-related LT [87, 89]. The prevalence of HCC in NASH-associated LT was higher compared with non-NASH-associated LT [78, 87, 88]. Interestingly, when subclassifying non-NASH-associated HCC on explant pathology, Agopian et al. showed a significantly higher prevalence of HCC in recipients with HCV and HBV, a similar prevalence in recipients with alcoholic liver disease and a lower prevalence in recipients with PBC/PSC when comparing with recipients with NASH [90]. Cardio/cerebrovascular events and infections are the leading cause of post-LT mortality in patients without concomitant HCC in both NAFLD and non-NAFLD-associated LT. Recurrent HCC is instead the most common cause of death in patients diagnosed with concomitant HCC, regardless of the cause of LT [77, 87]. The retransplantation rate was not different when comparing NAFLD and non-NAFLD recipients [90].

## NAFLD/NASH following liver transplantation

The development of recurrent and de novo NAFLD after liver transplantation is often diagnosed. Improvements in immunosuppressive therapies and surgical techniques in the past decades have increased long-term survival rates in patients receiving LT. As a result, chronic liver disease is more likely to occur after LT. It must be considered that while LT cures liver disease, it does not remove the risk factors for chronic liver disease. In particular, recipients who underwent LT due to NAFLD-associated HCC or ESLD still have the same risk factors (obesity, insulin resistance, hypertension, dyslipidaemia) that initially caused NAFLD before LT. Moreover, regardless of the cause, patients after LT are more susceptible to the development of MS, the main driver of NAFLD. De novo MS and de novo NAFLD mainly result from immunosuppressive therapy after LT. In addition to corticosteroids, calcineurin inhibitors and mammalian target of rapamycin inhibitors have been shown to trigger MS. The combination of pre-existing risk factors and immunosuppressive therapy is associated with a high risk of recurrence of NAFLD after LT. In addition, the genetic predisposition makes patients more susceptible to recurrence of steatosis. The presence of the allele rs738409-G of patatin-like phospholipase domain-containing protein 3 correlates with the recurrence of steatosis [59, 92–94].

The recurrence of NAFLD and NASH after LT is common; however, recurrence rates are controversial. Studies have shown more than 80% of NAFLD recurrence and 70% of NASH and fibrosis recurrence 5 years after LT. Recently, Saeed et al. showed in a meta-analysis an incidence of 82% of NAFLD recurrence and of 78% for de novo NAFLD 5 years after LT, respectively. Notably, the incidence of NASH recurrence was considerably higher when compared with de novo NASH 1 year after LT (53 vs 13%) [95]. In a retrospective study, Bhati et al. showed that the recurrence rates for NAFLD and NASH were 88.2% and 41.2%, respectively, when diagnosed by a liver biopsy 47 months median after LT [96]. Further studies have shown that NAFLD recurrences are more common than de novo NAFLD. In addition, the severity of steatosis and fibrosis is higher in NAFLD recurrence compared with de novo NAFLD [97–99]. Interestingly, Sourianarayanane et al. found a lower rate of fibrosis progression in patients with recurrent NASH compared with de novo NASH in patients transplanted for alcoholic liver disease [100]. A prevalence of de novo NAFLD ranging from 14.7 to 52% and of de novo NASH from 0.96 to 32% was reported by Losurdo et al. in a meta-analysis. Recipients with prior alcoholic liver disease showed the highest prevalence of de novo NAFLD [101]. The histologic recurrence pattern and de novo NAFLD appear to be similar but sufficient evidence is lacking [102]. Comparing the available data on the prevalence of NAFLD post-LT requires caution as the follow-up and NAFLD diagnostic methods are often different between studies. Variations in the patient cohort must also be taken into account when comparing different studies.

## Bariatric surgery

For NAFLD/NASH, lifestyle changes, in particular weight loss, are still the most important treatment strategies. No drug is currently available that significantly improves the course of the disease. However, patients do not always achieve sufficient weight loss and significant lifestyle changes. As a result, bariatric surgery has become an essential alternative for the treatment of severe NAFLD. The link between being overweight and developing MS is well known, and weight loss can be achieved through bariatric surgery. Weight loss improves both glucose homeostasis and fat metabolism, modulates intestinal hormones, reduces inflammatory activity, and consequently improves NAFLD [103, 104]. The most common techniques for bariatric surgery are sleeve gastrectomy, gastric bypass, and gastric banding. Several studies have shown positive results for NAFLD after bariatric surgery. Recently, von Schönfels et al. showed a significant improvement in NAFLD activity scores after bariatric surgery. Hereby, sleeve gastrectomy showed favorable results compared with Roux-en-Y gastric bypass [105]. Still, when sufficient weight loss was achieved, a reversal of NASH after Roux-en-Y gastric bypass was shown [106, 107]. A meta-analysis by Lee et al. investigating 32 cohort studies showed biopsy-confirmed resolution of steatosis in 66%, inflammation in 50%, and degeneration of ballooning in 76% of patients after bariatric surgery. The NAFLD activity score has also been reduced as well. Interestingly, after bariatric surgery, 12% of the patients showed new onset or worsening of NAFLD [108]. In addition, steatosis was resolved in 75%, lobular inflammation in 75%, chronic portal inflammation in 49%, steatohepatitis in 90%, and fibrosis in 53%. Remarkably, 2nd and 3rd degree fibrosis improved in 60% [109]. A meta-analysis by Mummadi et al. showed an improvement in steatosis in > 90%, steatohepatitis in > 80%, and fibrosis in > 60%. Complete resolution of NASH occurred in 69.5% [110]. In line with these results, Tan et al. demonstrated a significant improvement of NASH following bariatric surgery in a long-term study with a 10-year follow-up. Gastric banding was shown to be inferior to other bariatric techniques [111]. Supporting this, Lassailly et al. also showed an advantage of gastric bypass procedures over laparoscopic gastric banding. This study also found that in 85% of patients after bariatric surgery, NASH has subsided, with a higher proportion seen in patients with mild NASH compared with patients with severe NASH [112]. In view of the constellation of comorbidities in patients with NAFLD and the resulting high perioperative risk, the surgical technique must be carefully selected to minimize perioperative complications. The data indicate a more severe early deterioration of liver function after Roux-en-Y gastric bypass compared with sleeve gastrectomy [113]. Weingarten et al. showed that when laparoscopic bariatric surgery was performed, neither the presence of NASH nor its severity affected the complication rates [114]. Nevertheless, long-term survival was reduced in obese patients with NASH after bariatric surgery [115].

As can be seen, bariatric surgery has proven to be an important tool in achieving a successful treatment strategy for patients with severe NAFLD/NASH. Once patients and an appropriate surgical technique are carefully selected, bariatric surgery is as a safe alternative that will significantly improve NAFLD/NASH.

## Bariatric surgery in combination with liver transplantation

LT is often the only curative treatment for NAFLD/NASH patients with cirrhosis, HCC, and ESLD. While LT resolves the underlying liver disease, the associated risk factors are not eliminated. Yet, to improve outcomes, it is essential to address these risk factors. The method of treatment is primarily geared to weight loss, and although lifestyle modifications are often recommended, patients do not always achieve the desired weight. In contrast, bariatric surgery has been shown to have a significant impact on weight loss. In the last decades, a combination therapy of LT and bariatric surgery has proven to be a successful alternative for severe NAFLD. The optimal timing of bariatric surgery is an important issue that must be considered as this can be done before, during, or after LT. Bariatric surgery prior to LT is associated with a reduction of metabolic comorbidities at the time of LT. Nevertheless, there is an increased risk for patients because their liver function continues to be impaired. A surgical history with a recent major surgery such as bariatric surgery followed by LT is an important burden for the patient and increases the surgical risk. In addition, bariatric surgical complications can delay the rescue of LT. Performing bariatric surgery and LT at the same time lowers costs and reduces comorbidities after LT. However, the complexity of this procedure has been associated with a potentially higher risk of perioperative sepsis. Performing bariatric surgery after LT is a technically demanding procedure. Furthermore, immunosuppressive therapy hinders proper wound healing and increases the risk of infection [59, 116]. Malabsorption is a common post-operative complication associated with bariatric surgery and varies, among other factors, depending on the approach and the technique used. This must be carefully considered as a dysfunctional and altered intestinal absorption can impair the uptake of immunosuppressive drugs and can lead to an endangered liver graft [102].

A study by Lin et al. analyzing sleeve gastrectomy approximately 6 years at median post-LT demonstrated the safe feasibility of this procedure and a remarkable weight loss. Graft function and immunosuppression were not affected. The authors point out that complication rates due to more adhesions resulting from a prior LT operation can occur more frequent [117]. The Roux-en-Y gastric bypass procedure after LT has proven to be practical by Al-Nowaylati et al. and improved metabolic parameters including weight loss, blood sugar control, and high-density lipoprotein levels. In this study, the Roux-en-Y gastric bypass was performed approximately 27 months post-LT in median [118]. Simultaneous LT and gastric sleeve resection has shown favorable weight loss results and lower risk of metabolic complications post-LT compared with using non-invasive weight loss strategies before LT [119]. A study by Lin et al. investigating laparoscopic sleeve gastrectomy prior to solid organ transplantation (approximately 17 months in median prior to transplantation) showed an improvement of patient’s candidacy for subsequent organ transplantation [120].

Taken together, bariatric surgery is safe with favorable outcome when selecting patients carefully. Further randomized large cohort studies are still required to determine the optimal time for bariatric surgery and long-term outcomes.

## Conclusion

The global escalation of NAFLD prevalence is alarming and has quickly emerged as the most common form of chronic liver disease worldwide. The progression from steatosis to more severe forms, such a steatohepatitis, fibrosis, cirrhosis, and the development of HCC, correlates with liver and non-liver-related life-threatening complication. The metabolic disorders observed in patients with NAFLD are common in patients with cardiovascular disease and explain the patient’s comorbidity and high mortality rates. Childhood obesity rates have increased dramatically worldwide, as has the prevalence of obesity-related liver disease in this population. Pediatric NAFLD is a problem for the present because patient comorbidities are high, and for the future, as complications will occur earlier.

New mechanistic insights into steatosis, inflammation, and fibrosis in NAFLD have revolutionized the understanding that leads to disease progression. NAFLD is a multifaceted disease that involves the complex interaction between environmental and non-environmental factors. Interesting scenarios, such as those observed in patients after PD, have revealed in new settings and conditions that promote NAFLD development. This poses a new challenge for the health system: patient follow-up after PD must be done long term, and the constellation of metabolic comorbidities in post-PD/NAFLD patients is a real burden for the patients and for medical decisions.

The increasing prevalence of NAFLD/NASH will have a growing impact on surgery in the coming years. The presence of NAFLD itself and the associated comorbidities burden the pre-operative diagnostic and have a negative impact on the peri- and post-operative results. In addition, surgical needs are increasing exponentially in parallel with NAFLD/NASH rates as the disease progresses to more severe stages requiring surgery as the ultimate treatment. While weight loss is the most acceptable treatment for NAFLD management, surgery is an alternative for NAFLD/NASH patients who cannot achieve this through conservative strategies or who suffer from ESLD. The data has shown favorable results for NAFLD/NASH patients undergoing surgery, including LT, either for ESLD, HCC, or weight loss purposes. The dramatic increase in LT demand and the growing prevalence of NAFLD in the donor pool are imminent and threatening challenges in the near future. Prospective randomized multicenter studies are required to further determine current guidelines for the surgical treatment of severe NAFLD/NASH.
